# The effect of intrahepatic cholestasis in pregnancy combined with different stages of hepatitis B virus infection on pregnancy outcomes: a retrospective study

**DOI:** 10.1186/s12884-024-06460-9

**Published:** 2024-04-06

**Authors:** Qian Gao, Xuejiao Li, Li Wang, Xiaozhi Tan, Zhe Li, Chengfang Xu

**Affiliations:** Department of Obstetrics, the Third Affiliated Hospital of Sun Yat-Sen University, Guangdong, Guangzhou, 510630 China

**Keywords:** Intrahepatic cholestasis of pregnancy, Hepatitis B virus, Pregnancy outcomes

## Abstract

**Background and aims:**

To investigate the impact of intrahepatic cholestasis of pregnancy (ICP) with hepatitis B virus (HBV) infection on pregnancy outcomes.

**Methods:**

We selected 512 pregnant women, collected the data including maternal demographics, main adverse pregnancy outcomes and maternal HBV infected markers HBeAg and HBV-DNA loads status, then have a comparative analysis.

**Results:**

There were 319 solitary ICP patients without HBV infection (Group I) and 193 ICP patients with HBV infection. Of the latter, there were 118 cases with abnormal liver function(Group II) and 80 cases with normal liver function(Group III). All HBV-infected pregnant women with ICP were divided into hepatitis Be antigen (HBeAg)-positive group (102 cases) and HBeAg-negative group (91 cases), according to the level of the serum HBeAg status; and into high viral load group (92 cases), moderate viral load group (46 cases) and low viral load group (55 cases) according to the maternal HBV-DNA level. Group II had a higher level of serum total bile acids, transaminase, bilirubin as well as a higher percentage of premature delivery, neonatal intensive care unit (NICU) admission and meconium-stained amniotic fluid (MSAF) compared with the other two groups(*P* < 0.05), but there were no significant differences in the above indicators between the Group I and Group III. Among the HBV-infected patients with ICP, HBeAg-positive group had a higher level of serum transaminase, bilirubin and bile acid as well as earlier gestational weeks of delivery, lower birth weight of new-borns and a higher rate of NICU admission than HBeAg-negative group (*P* < 0.05). Those with a high viral load (HBV-DNA > 10^6^ IU/ml) had a higher level of transaminase, bilirubin, and bile acid as well as shorter gestational weeks of delivery, lower birth weight of new-borns and a higher rate of NICU admission compared with those with a low or moderate viral load (*P* < 0.05).

**Conclusion:**

HBV-infected pregnant women with ICP combined with abnormal liver function have more severe liver damage, a higher percentage of preterm birth and NICU admission. HBeAg-positive status and a high HBV-DNA load will increase the severity of conditions in HBV-infected pregnant women with ICP. HBV-infected patients with ICP who have abnormal liver function, HBeAg-positive or a high viral load should be treated more actively.

**Supplementary Information:**

The online version contains supplementary material available at 10.1186/s12884-024-06460-9.

## Background

Intrahepatic cholestasis during pregnancy (ICP) is a common complication of pregnancy, characterized by elevated bile acid levels, skin pruritus, and elevated transaminase levels in the second half of pregnancy. ICP can lead to complications such as preterm delivery, fetal distress, MSAF, and perinatal morbidity and mortality [1, 2].

Hepatitis B infection is caused by hepatitis B virus (HBV) and is a significant public health problem worldwide. There are nearly 87 million chronic HBV carriers in China, accounting for approximately one third of all chronic hepatitis B infection in the world [3]. According to a nationally representative serological survey in China in 2014, the prevalence of hepatitis B surface antigen (HBsAg) positivity in women of childbearing age is approximately 4.4%-5.9% [4].

The chronic HBV infection status has been traditionally characterized with four phases: immune-tolerant phase, HBeAg-positive immune-active phase, inactive CHB phase and HBeAg-negative immune reactivation phase. During pregnancy, immune imbalance, physiological and endocrine variation may lead to high replication of HBV, prompting hepatitis B activation or reactivation, resulting elevated ALT and high HBV DNA levels, and often accompanied with elevated total bile acid (TBA) and bile acids levels. However, it is unknown whether elevated bile acid levels differ between ICP and HBV infection and how they affect pregnancy outcomes.

## Patients and methods

### Study design and participant population

We conducted a retrospective study at the Third Affiliated Hospital of Sun Yat-Sen University from January 2011 to December 2021. All data were abstracted from the electronic medical records system and collected in the database using a standardized template.

### Inclusion criteria and exclusion criteria

According to ICP diagnostic criteria, patients with elevated serum TBA levels(≥ 10 μmol/L) were included [1]. HBV infection was defined as HBsAg seropositivity. Abnormal liver function was with alanine aminotransferase (ALT) level higher than 40 U/L.

Patients were excluded if they had any of the following situations: (1) had other pregnant complications, such as gestational hypertension, gestational diabetes mellitus, autoimmune disease, or renal disease; (2) had other hepatitis virus infections, including hepatitis A, C, D or E; (3) had other liver disease, including alcoholic hepatitis, autoimmune hepatitis, drug-induced hepatitis, or liver injuries caused by toxic substances or other causes; and (4) termination of pregnancy before 12 weeks.

### Selection of subjects

A total of 512 pregnant women were recruited, and all recruited subjects delivered in our hospital. Of these subjects, there are 319 ICP patients without HBV infection (Group I) and 193 ICP patients with HBV infection. According to the HBV infection phases and stages [5], we divided the ICP patients with HBV infection group into two groups: HBV infected women with abnormal liver function(*n* = 113, Group II) and those with normal liver function(*n* = 80, Group III). Of all the HBV-infected pregnant women with ICP, 102 had a positive HBeAg status and 91 had a negative HBeAg status according to the level of the serum HBeAg status; 92 patients were divided into high viral load group(> 10^6^ IU/mL), 46 patients were divided into moderate viral load group(10^3^–10^6^ IU/mL) and 55 patients were divided into low viral load group(< 10^3^ IU/mL) according to the maternal HBV-DNA level.

**Fig.1:**
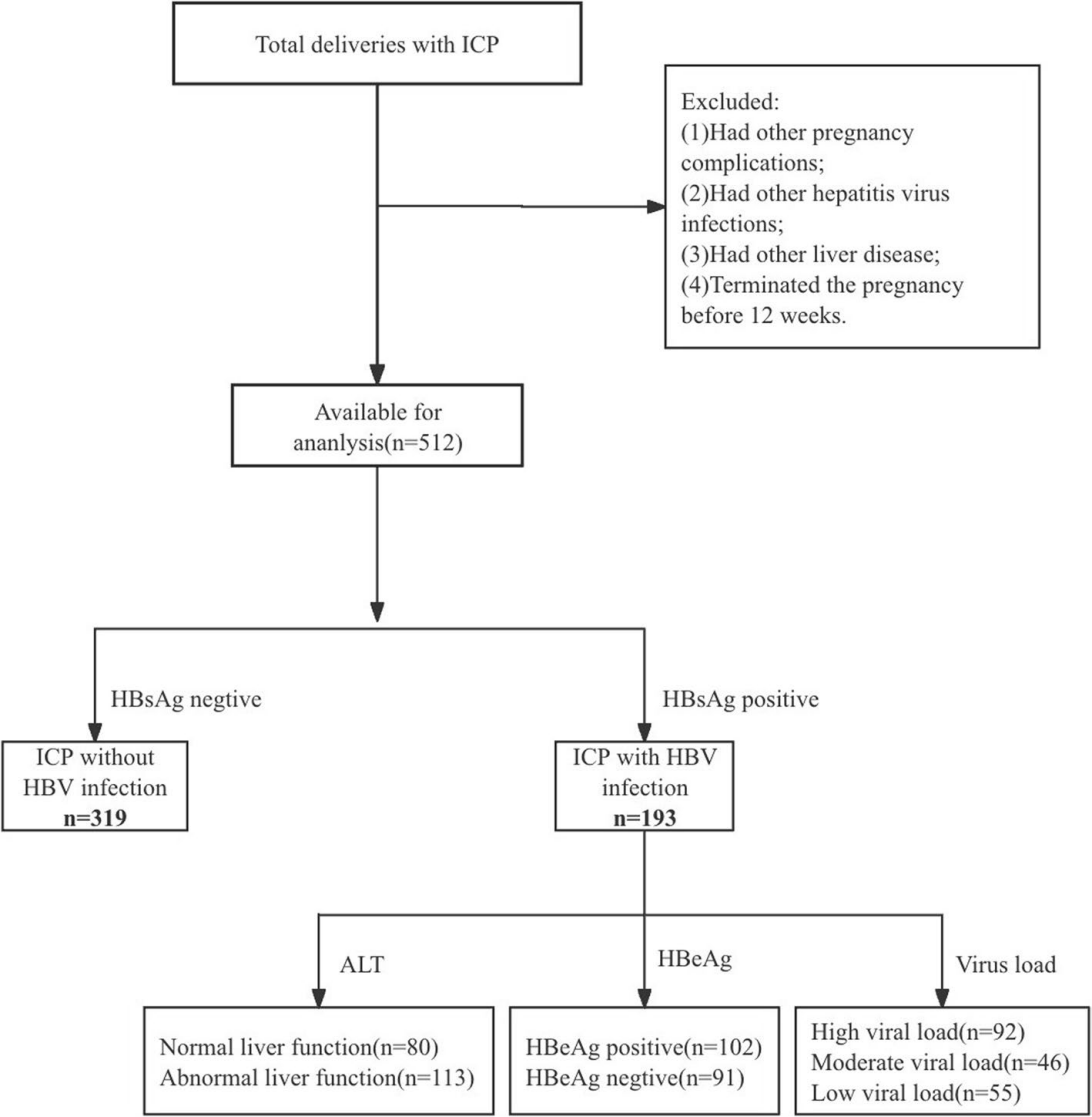
Flow chart for study population. ICP, intrahepatic cholestasis of pregnancy; HBV, hepatitis B virus; ALT, alanine transaminase

Maternal and neonatal demographic characteristics, including age, gestational age (weeks), birth weight (g), birth height (cm), Apgar score, and pregnancy outcomes, including preterm birth, admitted to the NICU and MSAF; medical history, antenatal laboratory data, maternal complications, and outcome data were extracted from the institutional medical record database. Figure 1 showed a flow chart of our study. (Fig. 1).

### Statistical analyses

Normally distributed continuous variables were presented as the means ± standard deviations (SD) and tested by analysis of variance or t-test; Non-normal distribution measurement variables were presented as median (interquartile range) and tested by Mann–Whitney U test or Kruskal–Wallis H test; Categorical variables are expressed as percentages and tested by Chi-square tests or Fisher’s exact tests. *P* values of < 0.05 were considered statistically significant. All of the statistical analyses were performed using the Statistical Program for Social Sciences (SPSS) 26.0 (Chicago, IL, USA) for Windows.

## Results

### Characteristics and adverse pregnancy outcomes of ICP, HBV-infected pregnant women with ICP combined with abnormal liver function and normal liver function

Table 1 summarized the demographic and clinical data among these three groups. As shown in Table 1, the patients in Group II had higher TBA, ALT, AST and TBIL levels than the other two groups and higher rates of preterm birth, MSAF, and admitted to the NICU (*P* < 0.05). Neonates of patients in Group II had lower 1-min Apgar scores (*P* < 0.05). The average gestational age in Group II was 35.81 ± 2.76 weeks, which was lower than that in the other two groups. There were no significant differences in liver function, bile acid and pregnancy outcomes between Group I and Group III (Table 1).

**Table 1:** Clinical characteristics of the different groups

	**Group I**	**Group II**	**Group III**
	**(ICP)**	**(ICP + HBV with abnormal liver function)**	**(ICP + HBV with normal liver function)**
Subjects (n)	319	113	80
Maternal age (years)	29.56 ± 4.27	30.1 ± 4.65	30.81 ± 4.7
Gestational age (weeks)	37.12 ± 2.38^a^	35.81 ± 2.76	37.31 ± 2.17^a^
Prepregnancy BMI (kg/m^2^)	20.36 ± 2.81	20.32 ± 2.35	20.31 ± 2.94
BMI at delivery (kg/m^2^)	25.03 ± 2.99	24.61 ± 2.77	24.81 ± 2.99
TBA (µmol/L)	37.65(25.13–53.69)^a^	71.5(47.39–123.03)	42.7(25.55–61.5)^a^
ALT (U/L)	14(10–29)^a^	96.25(276.5–554.75)	18(13–23.5)^a^
AST (U/L)	19(16–29)^a^	224(70–488.5)	21(16.75–27.25)^a^
TBIL (µmol/L)	7.15(5.1–10.1)^a^	12(7–25.7)	7(4.88–9.4)^a^
Preterm birth (n%)	117(36.7%)^a^	71(62.80%)	23(28.7%)^a^
MSAF (n%)	32(10.03%)^a^	34(30.09%)	9(11.25%)^a^
Birth height (cm)	47.14 ± 3.1	46.03 ± 3.16	47.72 ± 3.47
Birth weight (g)	2782.4 ± 560.3	2594.3 ± 527.6	2867.07 ± 512.2
Apgar score (1 min)	9.75 ± 0.9^a^	9.67 ± 1.06	9.93 ± 0.3^a^
Asphyxia neonatorum (n%)	6(1.88%)	2(1.77%)	0(0.0%)
Admitted to the NICU (n%)	155(48.59%)	86(76.11%)	32(40.00%)^a^
Intrauterine death (n%)	2(0.6%)	2(1.8%)	1(0.1%)

Normal distribution measurement data were expressed as mean ± SD and analyzed by analysis of variance. Non-normal distribution measurement data were expressed as median (interquartile range) and analyzed by Kruskal–Wallis H test; The frequency of preterm birth, MSAF, asphyxia neonatorum, admitted to the NICU and intrauterine death were compared by Chi-square tests or Fisher’s exact tests

*Abbreviations*: *BMI* body mass index, *TBA* total bile acid, *ALT* alanine transaminase, *AST* aspartate transaminase, *TBIL* total bilirubin, *MSAF* meconium-stained amniotic fluid, *NICU* neonatal intensive care unit

^*a*^*P* < 0.05, compared with Group II

### Associations of maternal HBeAg-positive status with adverse pregnancy outcomes among the HBV-infected pregnant women with ICP

To further evaluate the effect of HBeAg status on perinatal outcomes, we compared HBeAg-positive pregnant women with HBeAg-negative pregnant women among HBV-infected pregnant women with ICP (including Group II and Group III). Pregnant women with both ICP and HBeAg positivity (*n* = 102) had higher levels of TBA, ALT, AST and TBIL and a higher risk of admitted to the NICU than those with HBeAg negativity (*n* = 91) (*P* < 0.05), and their neonates had lower birth heights and birth weights (*P* < 0.05). In addition, there were 77 patients with ALT > 40U/L, accounting for 75.5%(77/102) in the patients with HBeAg-positive, and 31 patients with ALT > 40U/L accounting for 33%(31/91) in the patients with HBeAg-negative (*P* < 0.05). (Table 2).

**Table 2:** Clinical characteristics with respect to HBeAg status among HBsAg-positive pregnant women

	**HBeAg positivity**	**HBeAg negativity**	***P* value**
Subjects (n)	102	91	
Gestational age (weeks)	35.75 ± 2.83	36.43 ± 2.44	0.021
ALT (U/L)	187(44.5–421.5)	19(13–127.5)	0.000
AST (U/L)	136.35(32–361.75)	23(17.75–23.00)	0.000
ALT > 40U/L(*n*%)	77(75.5%)	31(33.0%)	0.000
TBIL (µmol/L)	10.25(6.13–19.85)	7.65(5.65–11.65)	0.015
TBA (µmol/L)	69.8(44–115.7)	46.4(26.8–46.4)	0.000
Preterm birth (*n*%)	46(45.1%)	32(35.2%)	0.160
MSAF (*n*%)	34(33.3%)	68(66.7%)	0.079
Birth height (cm)	46.33 ± 3.34	47.35 ± 3.01	0.038
Birth weight (g)	2596.52 ± 523.03	2840.24 ± 520.56	0.002
Apgar score (1 min)	9.70 ± 1.035	9.87 ± 0.56	0.195
Admitted to the NICU (*n*%)	70(68.6%)	48(52.7%)	0.024
Intrauterine death (*n*%)	2(2.0%)	1(1.1%)	0.629

Normal distribution measurement data were expressed as mean ± SD and analyzed by t test. Non-normal distribution measurement data were expressed as median (interquartile range) and analyzed by Mann–Whitney U test; The frequency of preterm birth, MSAF, asphyxia neonatorum, admitted to the NICU and intrauterine death were compared by Chi-square tests or Fisher’s exact tests

*Abbreviations:* *TBA*, total bile acid, *ALT* alanine transaminase, *AST* aspartate transaminase, *TBIL* total bilirubin, *MSAF* meconium-stained amniotic fluid, *NICU,* neonatal intensive care unit

### Associations of maternal HBV-DNA status with adverse pregnancy outcomes among the HBV-infected pregnant women with ICP

We performed a further analysis to determine whether a high HBV DNA load in HBV-infected pregnant women with ICP was associated with a higher risk of adverse pregnancy outcomes. The pregnant women in Group II and Group III were divided into three groups according to their HBV DNA load during pregnancy: those with a low viral load (< 10^3^ IU/mL), those with a medium viral load (10^3^–10^6^ IU/mL), and those with a high viral load (> 10^6^ IU/mL).Among all HBV-infected pregnant women with ICP, those with a high HBV DNA load (> 10^6^ IU/mL) had higher levels of TBA, ALT, AST and TBIL and higher risks of admitted to the NICU and MSAF than those with low viral load (< 10^3^ IU/mL), and their neonates had lower birth heights and weights (*P* < 0.05) (Table 3).

**Table 3:** Clinical characteristics with respect to the HBV DNA levels of HBsAg-positive pregnant women

	** < 10**^**3**^** IU/ml**	**10**^**3**^**–10**^**6**^** U/ml**	** > 10**^**6**^** IU/ml**
Subjects (n)	55	46	92
Gestational age (weeks)	36.65 ± 2.15	36.00 ± 3.00	35.66 ± 2.69^a^
Peak TBA level (µmol/L)	41.5(28.05–61.85)	53.4(30.63–115.03)	67.3(44–108.8)^a^
ALT (U/L)	16(10.5–21.5)	59.5(19–402.98)^a^	198(56–517)^a^
AST (U/L)	20(16–27.5)	50.5(23.5–365)^a^	181(41–430)^a^
TBIL (µmol/L)	6.8(4.65–8.85)	10.05(6.1–17.6)^a^	10.4(7.15–45.45)^a^
Preterm birth (*n*%)	15(27.3%)	22(47.8%)^a^	53(57.6%)^a^
MSAF (*n*%)	9(16.4%)	13(28.3%)	27(29.3%)^a^
Birth weight (g)	2795.42 ± 491.67	2787.73 ± 468.78	2600.94 ± 531.78^a^
Birth height (cm)	47.40 ± 3.36	47.16 ± 3.24	46.17 ± 3.28^a^
Apgar score (1 min)	9.92 ± 0.34	9.75 ± 0.611	9.64 ± 1.51
Admitted to the NICU (*n*%)	24(43.6%)	27(58.7%)^a^	62(67.4%)^ab^
Intrauterine death (*n*%)	1(1.8%)	0	2(2.2%)

Normal distribution measurement data were expressed as mean ± SD and analyzed by analysis of variance. Non-normal distribution measurement data were expressed as median (interquartile range) and analyzed by Kruskal–Wallis H test; The frequency of preterm birth, MSAF, asphyxia neonatorum, admitted to the NICU and intrauterine death were compared by Chi-square tests or Fisher’s exact tests

*Abbreviations*: *TBA* total bile acid, *ALT* alanine transaminase, *AST* aspartate transaminase, *TBIL* total bilirubin, *MSAF* meconium-stained amniotic fluid, *NICU* neonatal intensive care unit

^*a*^*P* < 0.05: Compared to the low viral load (< 10^3^ IU/mL) group; ^*b*^*P* < 0.05: Compared to the medium viral load (10^3^–10^6^ U/mL) group

## Discussion

ICP and HBV infection affect each other, interact with each other, and are closely related. Hepatitis B during pregnancy may represent acute or chronic infection or the reactivation of a prior infection, causing abnormal ALT and increased serum bile acid and bilirubin levels and leading to adverse pregnancy outcomes, similar with the biochemical characteristics of ICP. However, it is difficult to distinguish whether these clinical manifestations in pregnant women with HBV infection are caused by the reactivation of HBV or by the occurrence of ICP in middle and late pregnancy. The pathogenesis of ICP may be related to the interaction of genetic susceptibility [6], altered estrogen and progesterone levels during pregnancy [7, 8], abnormalities in the body's immune system, and environmental factors, but the pathogenesis of HBV with ICP has not yet been reported and fully understood. Our study is the first to report pregnant outcomes of ICP and ICP combined with HBV infection with different maternal liver function, and further explore the influence of ICP with HBeAg and HBV DNA status on adverse pregnancy outcomes.

Several studies have shown that HBV infection is linked to an increased risk of ICP [9–11]. A recent meta-analysis from 2020 [9] on the relationship between ICP and HBV infection found an increased risk of ICP among HBV-infected pregnant women. In that study, the odds ratio (OR) of ICP in HBV-infected pregnant women compared with non-HBV-infected pregnant women was 1.68 (95% CI 1.43–1.97). A large cross-sectional retrospective study in China found that women with HBsAg-positive status showed an increased risk of ICP, OR was 2.79 (95% CI 2.36–3.30) [10]. However, these studies did not report pregnancy outcomes between ICP with or without HBV infection. Another study [12] showed that ICP with HBV infection had more serious effects on newborns, aggravated ICP and virus infection symptoms in mothers. In this study, ICP with HBV infection patients had more adverse fetal outcomes including birth defects, fetal distress and neonatal asphyxia. We observed higher maternal ALT, AST, serum bile acid and bilirubin levels in HBV infected women with abnormal liver function, as well as adverse pregnancy outcomes such as MSAF, preterm delivery, low birth weight and admitted to the NICU (*P* < *0.05*) (Table 1), which is consistent with the previous studies [12, 13]. The advantage of our study is that we observed more comprehensive maternal and fetal outcomes and our sample size was larger. We also investigated the differences in pregnancy outcomes among liver dysfunction, biliary tract dysfunction, cholestasis and the difference between elevated TBA due to HBV activation and solitary TBA elevation. In addition, we found that there was no significant difference in pregnancy outcomes between HBV-infected pregnant women with ICP combined with normal liver function and solitary ICP (*p* > 0.05), but adverse pregnancy outcomes such as liver injury, the rate of admitted to NICU and MSAF were more likely to occur in HBV-infected patients with ICP combined with abnormal liver function (*p* < 0.05). Although the exact mechanism of the relationship between HBV infection and the progression of ICP is unclear, there are a few possible explanations. A possible explanation is that Hepatitis B virus infection causes an autoimmune reaction, ultimately leading to serious negative effects such as liver cell damage and placental dysfunction, increased cholestasis, significant hypoxia, fetal distress, premature birth or stillbirth. Several studies have shown that HBV infection is associated with oxidative stress, and oxidative stress plays an important role in the development of ICP. So ICP patients with HBV-infection and liver injury may have an increased oxidative stress resulted in significantly worse pregnancy outcomes [14].

Our study also examined the effects of ICP with HBeAg and HBV DNA status on adverse pregnancy outcomes. Women of childbearing age who are infected with HBV are generally relatively young, and most of them are in the immune tolerance period. In this immune tolerance period, body of HBV patients were predominated of HBeAg-specific Th2 cells, inducing Th1/ Th2 imbalance. Many studies have reported a predominant Th2-type immunity and suppressed Th1-type immunity during pregnancy. This immunity status is more beneficial for fetus growth without rejection reaction. Compared with non-pregnant women, pregnant women have more Th2-type inflammatory factor [15]. This imbalance status will induce HBV infection progression. Meanwhile, physiological variation, metabolic rate elevating and more liver burden lead to high replication of HBV, prompting hepatitis B activation or reactivation, resulting in liver function abnormalities.

A study in pregnant North American women with chronic hepatitis B infection found that among the 158 pregnant women with chronic HBV, serum ALT flares developed in 3.4% during pregnancy [16]. Another study in Nanjing City of China found that there were 72 patients with HBeAg-positive immune active, accounting for about 20.2% (72/356) in the patients with HBeAg-positive, and 61 patients with HBeAg-negative immune active, accounting for about 9.9% (61/356) in the patients with HBeAg-negative [17]. In our study, We found a much higher proportion(75.5%)of abnormal liver function among HBeAg-positive patients with ICP(77/102). This results might be related to the reason that our study population is HBV infection combined with ICP. Compared with pregnant women with solitary hepatitis B infection, the proportion of abnormal liver function is higher. We found that ICP patients with HBeAg positivity and high HBV-DNA viral load had a higher rate of adverse pregnancy outcomes, such as preterm birth, admitted to the NICU and MSAF as well as more severe liver injury, such as elavated ALT, TBA and TBIL. Cai et al. [18] found that HBeAg positivity is associated with a higher risk of ICP, means that HBeAg positivity aggravates ICP and lead to higher levels of bile acids than in HBeAg-negative pregnant women. Ju et al. [17] found that patients in the HBeAg positive or immune active phases had a higher incidence of ICP and the preterm birth rate. HBeAg may increase the risk of ICP by affecting bile acid metabolism [19]. However, the research on HBeAg and ICP is limited, and more studies are needed.

Maternal HBeAg positive and a high HBV DNA viral load are considered markers of HBV replication and disease severity [20], they also might aggravate the inflammatory response, which is a mediator of ICP [21, 22] and increases the risk of pregnancy complications and adverse neonatal outcomes [23, 24]. We mentioned above that oxidative stress is involved in the pathogenesis of ICP, HBV is a kind of hepatophilic virus that has been related to the development of oxidative stress. It is believed that HBV generates oxidative stress by altering mitochondrial function and modulating host gene expression. Another study further revealed that HBV virus altered the expression profile of bile acid metabolism genes by binding to cellular receptors [25]. HBV infects the placenta, alters the intrauterine environment and changes the inflammatory response of the placenta, which causes respiratory, metabolic and nutritional insufficiency of the placenta and might contribute to preterm birth [19]. The higher preterm birth rate may account for the higher NICU admission rate and the lower birth weights and heights. That might be why the higher HBV DNA viral load with ICP have a significant negative impact on pregnancy outcomes. However, Cheung et al. reported that a seropositive HBeAg status or a higher HBV DNA viral load during pregnancy did not have a significant negative impact on pregnancy outcomes [26]. This results might due to that HBeAg was tested at recruitment time, but HBV DNA level was quantified at 28–30 weeks of gestation. Women with antiviral treatment during pregnancy for their high viral load or absence of HBV DNA result at 28–30 weeks for occurrence of premature delivery were excluded. This exclusion induced some different results with our study.

There are some limitations in this study. Firstly, in this single center retrospective study, we could not exclude selection and information bias. Secondly, we only analyzed maximum TBA levels and we did not determine the TBA levels after delivery. Thirdly, our study was limited in taking antiviral treatment into account since the data on antiviral treatments were not completely recorded. Further clinical trial should be conduct for more detail investigation in TBA levels and other complications.

## Conclusion

Our study confirmed that ICP combined with HBV-infected patients with abnormal liver function have more severe liver damage, a higher percentage of preterm birth and NICU admission. HBeAg-positive status and a high HBV-DNA load will increase the severity of conditions in HBV-infected pregnant women with ICP. But we did not find any significant differences in pregnancy outcomes between HBV-infected pregnant women with ICP combined with normal liver function and the group of solitary ICP. In clinical practice, the management of the perinatal period of former patients is recommended to follow the principles of management for ICP; and patients with ICP who have abnormal liver function, HBeAg-positive or a high viral load should be treated more actively.

### Supplementary Information

**Supplementary Materials 1.**

## Data Availability

Data is provided within supplementary information files.

## Abbreviations

MSAF: Meconium-stained amniotic fluid
ICP: Intrahepatic cholestasis of pregnancy
HBV: Hepatitis B virus
CHB: Chronic hepatitis B
HBeAg: Hepatitis B e antigen
NICU: Neonatal intensive care unit
HBsAg: Hepatitis B surface antigen
ALT: Alanine transaminase
AST: Aspartate transaminase
TBA: Total bile acid
TBIL: Total bilirubin
BMI: Body mass index
OR: The odds ratio
